# Supplementary material for: Accelerating Medicines Partnership: Parkinson's Disease. Genetic Resource
Source: Mov Disord. 2021 May 7;36(8):1795–804. doi: 10.1002/mds.28549 (PMC8453903; doi:10.1002/mds.28549)
Supplement: Supplementary file 1 — Appendix S1. Supplementary Information [file MDS-36-1795-s001.pdf]

Supplemental Figure 1. Population plots with references populations

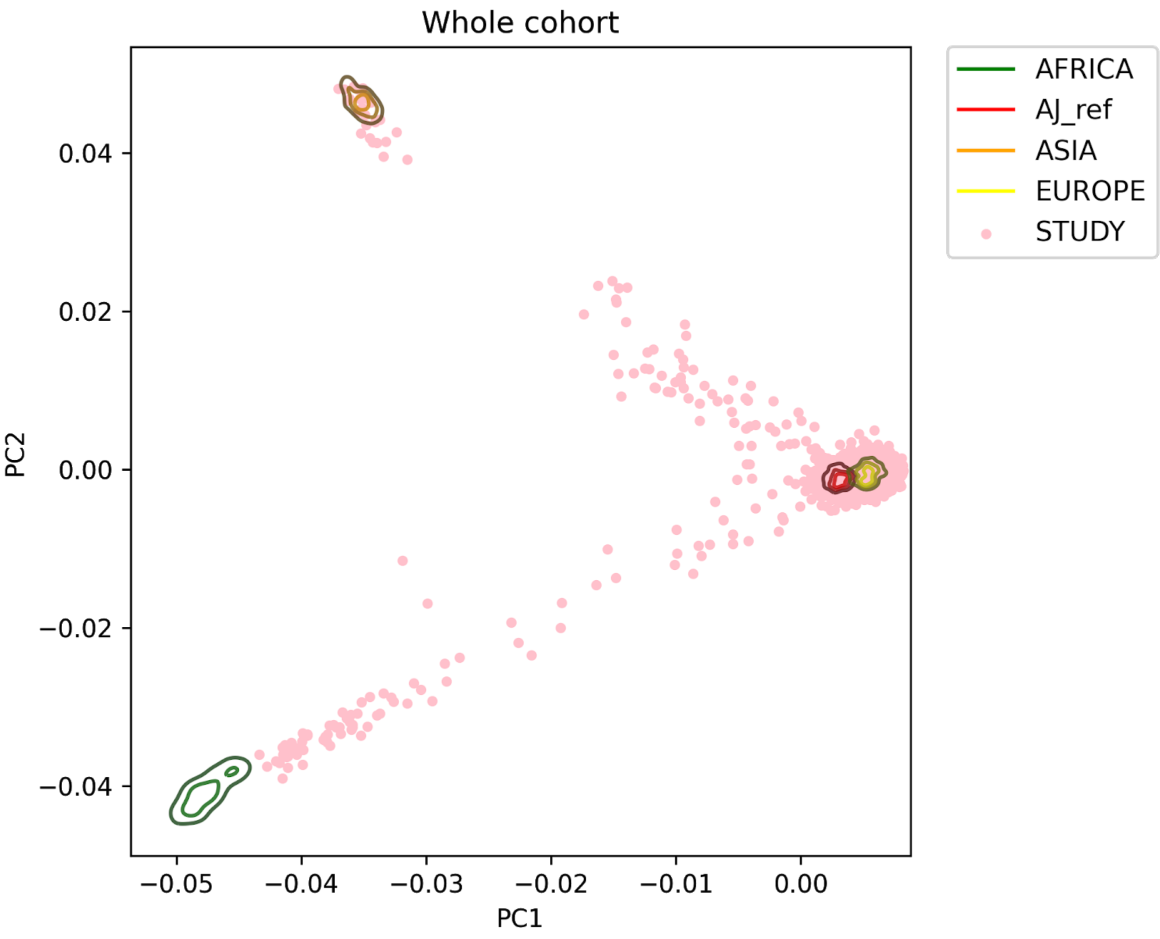

Supplemental Figure 2. Genetic PC plots of European ancestry individuals

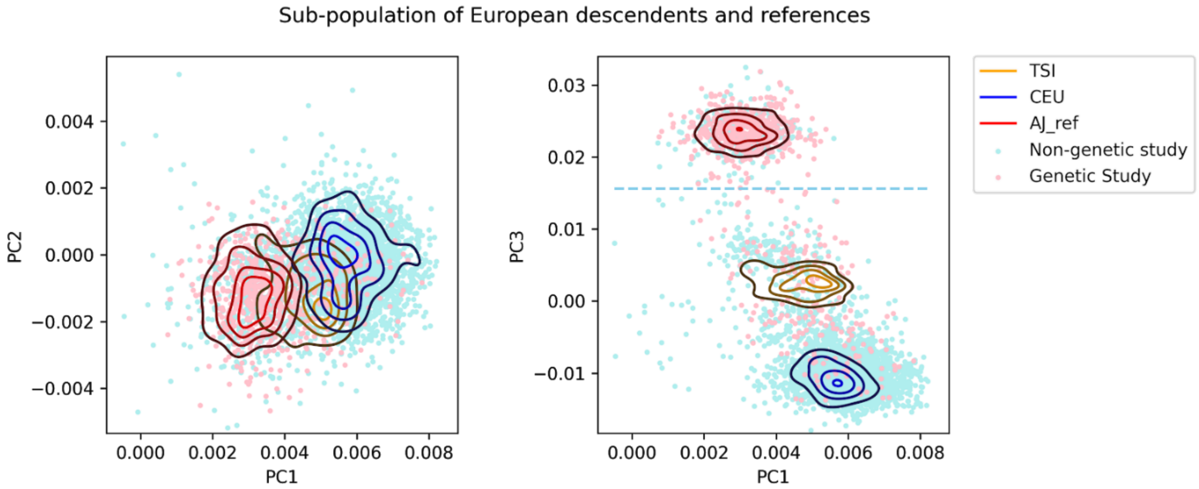

Blue dashed line represents the cut-off value to infer AJ population
AJ_ref, Ashkenazi Jewish reference panel; CEU Northern Europeans from Utah; TSI, Toscani from Italy. Genetic study, genetically enriched cohorts; Non-genetic study, not genetically enriched cohorts.

Supplemental Table. AJ percentage among Europeans

Cohort	Category	N of Europeans	AJ %
BioFIND			
	HC	70	14.3
	PD	99	16.2
HBS			
	HC	227	11.9
	PD	640	12.3
PDBP			
	HC	470	3.2
	PD	856	3.2
PPMI Original Cohort			
	HC	190	11.1
	PD	408	5.4
	SWEDD	61	6.6
PPMI Prodromal Cohort			
	Prodromals	61	3.3
PPMI Genetic Cohort			
	HC_GC	210	72.4
	PD_GC	178	53.9
PPMI Genetic Registry			
	HC_GR	164	67.7
	PD_GR	125	85.6
All		3759	18.3

AJ, Ashkenazi Jewish; BF, BioFIND; PD, PDBP; PP, PPMI; HC, Healthy controls; PD, Parkinson's disease; GC, Genetic Cohort; GR, Genetic Registry.

Group Authorships

Name	Affiliation	AMP PD WGS Role
Andrew B Singleton	Center for Alzheimer's and Related Dementias, National Institute on Aging, Bethesda, MD, USA<br><br>Laboratory of Neurogenetics, National Institute on Aging, Bethesda, MD, USA	Co-chair
Ashutosh Pandey	GlaxoSmithKline, 9911 Belward Campus Dr, Rockville, MD 20850, USA	Member
Barry Landin	Technome, Herndon, VA, USA	Member
Bradford Casey	The Michael J. Fox Foundation for Parkinson's Research, New York, NY, USA	Member
Christine Swanson-Fischer	National Institute of Neurological Disorders and Stroke, National Institutes of Health, Bethesda, MD 20824, USA	Member
Clemens R. Scherzer	Harvard Medical School, Brigham and Women's Hospital, Boston, MA, USA	Member
David Pulford	GlaxoSmithKline, 9911 Belward Campus Dr, Rockville, MD 20850	Member
David Vismer	Technome, Herndon, VA, USA	Member
Debra Babcock	National Institute of Neurological Disorders and Stroke, National Institutes of Health, Bethesda, MD 20824, USA	Member
Dena G Hernandez	Laboratory of Neurogenetics, National Institute on Aging, Bethesda, MD, USA	Member
Dinesh Kumar	Sanofi, Seattle, WA, USA	Member
Dongyu Liu	Sanofi, Seattle, WA, USA	Member
Eline Appelmans	11400 Rockville Pike #600, North Bethesda, MD 20852, USA	Facilitator
Hampton L. Leonard	Data Tecnica International, Glen Echo, MD, USA<br><br>Center for Alzheimer's and Related Dementias, National Institute on Aging, Bethesda, MD, USA<br><br>Laboratory of Neurogenetics, National Institute on Aging, Bethesda, MD, USA	Member
Hirotaka Iwaki MD	Data Tecnica International, Glen Echo, MD, USA	Member

	Center for Alzheimer's and Related Dementias, National Institute on Aging, Bethesda, MD, USA<br><br>Laboratory of Neurogenetics, National Institute on Aging, Bethesda, MD, USA	
J. Raphael Gibbs	Laboratory of Neurogenetics, National Institute on Aging, Bethesda, MD, USA	Member
Lynn Jakeman	National Institute of Neurological Disorders and Stroke, National Institutes of Health, Bethesda, MD 20824, USA	Member
Mahdiar Sadeghi	The American Genome Center, Uniformed Services University of the Health Sciences, Bethesda, MD, USA<br><br>Previously Sanofi, Seattle, WA, USA	Alumni
Mary B. Makarious	Laboratory of Neurogenetics, National Institute on Aging, Bethesda, MD, USA	Member
Margaret Sutherland	801 Jefferson Avenue, Redwood, CA, USA<br><br>Previously National Institute of Neurological Disorders and Stroke, National Institutes of Health, Bethesda, MD 20824, USA	Alumni
Mark Frasier	The Michael J. Fox Foundation for Parkinson's Research, New York, NY, USA	Member
Matt Edwards	Verily Life Sciences, San Jose, CA, USA	Member
Matt Bookman	Verily Life Sciences, San Jose, CA, USA	Member
Meaghan Cogswell	Sanofi, Seattle, WA, USA	Member
Mike A. Nalls	Data Tecnica International, Glen Echo, MD, USA<br><br>Center for Alzheimer's and Related Dementias, National Institute on Aging, Bethesda, MD, USA<br><br>Laboratory of Neurogenetics, National Institute on Aging, Bethesda, MD, USA	Member
Robert Moccia	1275 Pennsylvania Avenue NW STE 600, Washington, DC 20004, USA	Member

Rosa Canet-Aviles	35 Cambridge Park Dr Suite 200, Cambridge, MA 02140<br><br>Previously 11400 Rockville Pike #600, North Bethesda, MD 20852, USA	Alumni
Shameek Biswas	Bristol Myers Squibb, Seattle, WA, USA	Co-chair
Sonja W. Scholz	National Institute of Neurological Disorders and Stroke, Bethesda, MD, USA<br><br>Department of Neurology, Johns Hopkins University, Baltimore, MD, USA	Member
Srini Shankara	Sanofi, Seattle, WA, USA	Member
Xianjun Dong	Harvard Medical School, Brigham and Women's Hospital, Boston, MA, USA	Member

AMP PD consortium (admin@amp-pd.org) members

Uniformed Services University of the Health Sciences Associates

Name	Affiliation	Title and Role
Adelani Adeleye	Henry M. Jackson Foundation for the Advancement of Military Medicine, Inc., Bethesda, MD 20817	System Administrator, implemented and conducted data transfer to NIH
Camille Alba	Henry M. Jackson Foundation for the Advancement of Military Medicine, Inc., Bethesda, MD 20817	Research Assistant, managed project and sample tracking, supervised and conducted DNA quality control, library preparation, library quality control, sequencing for WGS
Dagmar Bacikova	Henry M. Jackson Foundation for the Advancement of Military Medicine, Inc., Bethesda, MD 20817	Lab Manager, manages laboratory logistics and supplies for team personnel
Clifton L. Dalgard	Department of Anatomy, Physiology & Genetics, Uniformed Services University of the Health Sciences, Betheda, MD 20814	Director, designed and directs all programmatic, laboratory and financial components for center activities
Daniel N. Hupalo	Henry M. Jackson Foundation for the Advancement of Military Medicine, Inc., Bethesda, MD 20817	Computational Biologist, oversaw WGS data quality control and assembled data transfer selection and tracking
Elisa McGrath Martinez	Henry M. Jackson Foundation for the Advancement of Military Medicine, Inc., Bethesda, MD 20817	Research Assistant, conducted library preparation, quality control, pooling and sequencing for WGS
Sraavya Polisetti	Henry M. Jackson Foundation for the Advancement of Military Medicine, Inc., Bethesda, MD 20817	Research Technician, performed DNA quality assessment, quality control and sequencing for WGS

John Rosenberger	Henry M. Jackson Foundation for the Advancement of Military Medicine, Inc., Bethesda, MD 20817	Senior Research Assistant, oversaw sequencing platform performance for WGS
Anthony R. Soltis	Henry M. Jackson Foundation for the Advancement of Military Medicine, Inc., Bethesda, MD 20817	Senior Computational Biologist, reviewed and evaluated WGS analysis pipeline and performance
Gauthaman Sukumar	Henry M. Jackson Foundation for the Advancement of Military Medicine, Inc., Bethesda, MD 20817	Research Associate, conducted library preparation, quality control, pooling and sequencing for WGS
Miranda F. Tompkins	Henry M. Jackson Foundation for the Advancement of Military Medicine, Inc., Bethesda, MD 20817	Research Technician, performed DNA quality assessment, quality control and sequencing for WGS
Meila Tuck	Henry M. Jackson Foundation for the Advancement of Military Medicine, Inc., Bethesda, MD 20817	Research Technician, performed DNA quality assessment, quality control and sequencing for WGS
Coralie Violett	Henry M. Jackson Foundation for the Advancement of Military Medicine, Inc., Bethesda, MD 20817	Scientist, conducted entry of subcohorts into project workload, initiated and tracked laboratory subprojects, reported subcohort quality control and completion
Matthew D. Wilkerson	Henry M. Jackson Foundation for the Advancement of Military Medicine, Inc., Bethesda, MD 20817	Bioinformatics Director, designed and directs WGS analysis pipeline and performance
Xijun Zhang	Henry M. Jackson Foundation for the Advancement of Military Medicine, Inc., Bethesda, MD 20817	Computational Biologist, designed and implements sequencing quality control dashboard and implemented panel-based cohort VCF generator
